# A Rat Model of Human Behavior Provides Evidence of Natural Selection Against Underexpression of Aggressiveness-Related Genes in Humans

**DOI:** 10.3389/fgene.2019.01267

**Published:** 2019-12-13

**Authors:** Dmitry Oshchepkov, Mikhail Ponomarenko, Natalya Klimova, Irina Chadaeva, Anatoly Bragin, Ekaterina Sharypova, Svetlana Shikhevich, Rimma Kozhemyakina

**Affiliations:** ^1^Institute of Cytology and Genetics, Siberian Branch of Russian Academy of Sciences, Novosibirsk, Russia; ^2^Natural Science Department, Novosibirsk State University, Novosibirsk, Russia

**Keywords:** behavior, rat model, aggressiveness, domestication, differentially expressed gene, hypothalamus, periaqueductal gray

## Abstract

Aggressiveness is a hereditary behavioral pattern that forms a social hierarchy and affects the individual social rank and accordingly quality and duration of life. Thus, genome-wide studies of human aggressiveness are important. Nonetheless, the aggressiveness-related genome-wide studies have been conducted on animals rather than humans. Recently, in our genome-wide study, we uncovered natural selection against underexpression of human aggressiveness-related genes and proved it using F1 hybrid mice. Simultaneously, this natural selection equally supports two opposing traits in humans (dominance and subordination) as if self-domestication could have happened with its disruptive natural selection. Because there is still not enough scientific evidence that this could happen, here, we verified this natural selection pattern using quantitative PCR and two outbred rat lines (70 generations of artificial selection for aggressiveness or tameness, hereinafter: domestication). We chose seven genes—*Cacna2d3*, *Gad2*, *Gria2*, *Mapk1*, *Nos1*, *Pomc*, and *Syn1*—over- or underexpression of which corresponds to aggressive or domesticated behavior (in humans or mice) that has the same direction as natural selection. Comparing aggressive male rats with domesticated ones, we found that these genes are overexpressed statistically significantly in the hypothalamus (as a universal behavior regulator), not in the periaqueductal gray, where there was no aggressiveness-related expression of the genes in males. Database STRING showed statistically significant associations of the human genes homologous to these rat genes with long-term depression, circadian entrainment, Alzheimer’s disease, and the central nervous system disorders during chronic *IL-6* overexpression. This finding more likely supports positive perspectives of further studies on self-domestication syndromes.

## Introduction

Ethologists define aggressiveness as a hereditary behavioral pattern important for preservation of the species. It is associated with the establishment of a social hierarchy in society and contributes to an increase in the individual reproductive potential (Lorenz, 2002). Because the individual social rank affects both quality and duration of life (Michopoulos et al., 2012), the genome-wide studies on human aggressiveness are vitally important. Yet, this research is still performed on laboratory animals: mice (Kudryavtseva et al., 2017), rats (Alttoa et al., 2010), rabbits (Albert et al., 2012), pigs (Zhou et al., 2014), dogs (Zapata et al., 2016), foxes (Kukekova et al., 2018), chickens (Natt et al., 2012), and trout (Christie et al., 2016), without the reference human genome (Telenti et al., 2016).

The reference human genome is represented by the databases Ensembl (Zerbino et al., 2015) [as a summary of over 10^4^ individual genomes (Telenti et al., 2016)], dbSNP (Sherry et al., 2001) [as over 10^8^ single-nucleotide polymorphisms (SNPs)], and dbWGFP (Wu et al., 2016) as data on all the 10^10^ possible SNPs intended for personalized medicine (Trovato, 2014). The basic concept of personalized medicine is a clinical SNP marker of human diseases identified as the statistically significant difference in the occurrence of its alleles within the representative cohorts of patients versus relatively healthy volunteers [see, e.g. (Varzari et al., 2018)]. Because these markers affect diagnosis and treatment of humans, they need to be studied, but the conventional way to identify and validate each of the 10^10^ possible human SNPs will be prohibitively expensive. Because of neutrality of the majority of human SNPs (Haldane, 1957; Kimura, 1968), the genome-wide search for and discarding neutral SNPs before identification of the clinical SNP markers can reduce the cost of this research.

Earlier, we created Web service SNP_TATA_Comparator^1^ for genome-wide SNP analysis (Ponomarenko et al., 2015). Using it, we predicted candidate SNP markers of human aggressiveness (Chadaeva et al., 2016) and natural selection against underexpression of human aggressiveness-related genes (Chadaeva et al., 2017). Recently, we proved this natural selection pattern using F1 hybrid male mice as an animal model of human heredity (Chadaeva et al., 2019). Simultaneously, this natural selection equally supports two opposing traits in humans (i.e., dominance and subordination) as if self-domestication could have happened with its characteristic disruptive natural selection as Belyaev (1979) discovered in wild and domesticated foxes half a century ago.

Because there is still not enough scientific evidence that this human self-domestication could happen, here, we verified this natural selection pattern using quantitative PCR (qPCR) and two outbred rat lines selected artificially for aggressiveness or tameness (hereinafter: domestication). We discuss the results as evidence either in favor or against recently discovered self-domestication syndrome (Theofanopoulou et al., 2017).

## Materials and Methods

### Study Design

According to many studies, various phenomena can affect gene expression during artificial selection, e.g., pleiotropic and epistatic effects, co-expression of the genes affecting target traits and the genes neutral toward them as well as stress-induced epigenetic reprogramming of genes that is inheritable for the next several generations as an expression modulator of these genes. Thus, nothing can be claimed about changes in the expression of any given gene during artificial selection, with the only exception when both artificial and natural selection change the expression of this gene in the same known direction, which becomes the actual direction of its expression change. Thus, taking advantage of the natural selection against underexpression of aggressiveness-related genes, using PubMed (Lu, 2011), we chose the genes to be analyzed whose over- or underexpression corresponds to aggressive or nonaggressive (hereinafter: domesticated) behavior in various animal models of human behavior.

Additionally, for a comparison between aggressive and domesticated male rats in the expression of these genes, we used only the hypothalamus–periaqueductal gray (PAG) pair as described elsewhere (Gorinski et al., 2019). We chose the hypothalamus as a universal brain region most often used in the studies on aggressiveness of female and male animals of all ages [see, e.g. (Walker et al., 2016)], while PAG is specific for both postpartum sexual and maternal aggressiveness (Lonstein and Stern, 1998), which are both absent in the male rats being analyzed here.

Accordingly, we heuristically predicted significant overexpression of the analyzed genes in the hypothalamus, but not PAG, of the aggressive male rats in comparison with the domesticated ones. Because it is still unknown whether this pattern is true, we tested this prediction by qPCR.

### Animals

The study was performed on adult male gray rats (*Rattus norvegicus*) selectively bred for over 70 generations for aggressive or domesticated behavior (as two outbred lines) under standard conditions of the Conventional Animal Facility of the Institute of Cytology and Genetics (Novosibirsk, Russia) as described elsewhere (Belyaev and Borodin, 1982; Naumenko et al., 1989; Plyusnina and Oskina, 1997).

The total number of rats was 12: six aggressive and six domesticated, each weighing 250–270 g, 4 months old, all from different unrelated litters. All the rats were decapitated. Following a handbook (Paxinos and Watson, 2013), we extracted samples of the hypothalamus and PAG, which were then flash-frozen in liquid nitrogen and stored at −70°C until use. Every effort was made to minimize the number of animals studied and their suffering.

All the procedures were in line with Directive 2010/63/EU of the European Parliament and of the European Council of September 22, 2010. The research protocol was approved by the Interinstitutional Commission on Bioethics at the Institute of Cytology and Genetics of the Siberian Branch of the Russian Academy of Sciences.

The **Supplementary File** “Supplementary experiment” describes these procedures in more detail.

### RNA Extraction and qPCR

From each above-mentioned sample of either the hypothalamus or PAG from 12 male rats, total RNA was extracted with the TRIzol™ Reagent (Invitrogen, cat. #15596018) and TURBO DNA-free™ Kit (Ambion, #AM1907). Total RNA was quantified on an Invitrogen Qubit™ 2.0 fluorometer (Invitrogen–Life Technologies). Integrity of total RNA was verified by means of Agilent Bioanalyzer 2100 (Agilent). cDNA was synthesized with the Phusion RT-PCR Kit (Thermo Scientific). Using PrimerBLAST, oligonucleotide primers for qPCR were designed (Table 1). qPCR was carried out by means of the EVA Green I Kit (Syntol, #R-441)) at least twice for each sample on a CFX-96 Touch System (Bio-Rad, USA), whose output was mean ± standard error of the mean (SEM) in arbitrary units as a gene expression metric. *Rpl30* (ribosomal protein L30) served as a reference gene.

**Table 1:** The aggressiveness-related genes chosen to be analyzed: primers and justification.

*Gene, OMIM ID*	Primers: 5′→3′ direct (D) and reverse (R)	Justification for choice of genes for qPCR		
		Species	Behavioral feature	References
*Cacna2d3, 606399*	D: taagctgcgacgatgagactg R: tgacagctccttcgacctca	humans	*Cacna2d3* deficiency induces mood and anxiety disorders	Baeza-Richer et al., 2015; Kassir, 2017
*Gad2, 138275*	D: gctcatcgcattcacgtcag R: ggcactcaccaggaaaggaa	mice	knockout of *Gad2* leads to sensitized pain behavior	Zhang et al., 2011
*Gria2, 138247*	D: ggactaccgcagaaggagtag R: aggccttgttcattcagttttagt	mice	increased expression of *Gria2* initiates a response to fear	Liu et al., 2010
*Mapk1, 176948*	D: caggttgttcccaaacgctg R: gagcccttgtcctgaccaat	humans	a microdeletion of *Mapk1* can increase risks of anxiety disorders	Verhoeven et al., 2011
*Nos1, 608226*	D: acccgacctcagagacaact R: aagcttcttcctgtccgcaa	mice	*Nos1* underexpression reduces maternal aggression	Gammie and Nelson, 1999
*Pomc, 176830*	D: catcatcaagaacgcgcacaa R: taactctaagaggctggaggtca	humans	anxiety and POMC levels correlate negatively in alcoholics	Kiefer et al., 2002
*Syn1, 313440*	D: tgccaatggtggattctccg R: cagcccaatgaccaaactgc	mice	*Syn1* underexpression induces anxiety behavior after early life stress	Park et al., 2014

Cacna2d3, Ca-channel α2δ3 subunit; Gad2, glutamate decarboxylase 2; Gria2, glutamate receptor 2; Mapk1, mitogen-activated protein kinase 1; Nos1, NO synthase 1; Pomc, proopiomelanocortin; Syn1, synapsin-1.

### Statistical Analysis

For each gene being analyzed, we converted the qPCR data (mean ± SEM) into two equivalent Dn and Up statistics corresponding the lower and upper boundaries of the [mean ± standard deviation] interval of these data (**Table S**, hereinafter: see **Supplementary file** “Supplementary experiment”), which passed both nonparametric and parametric tests, while neither the mean nor the SEM information is lost.

We conducted the Kolmogorov–Smirnov test as well as Fisher’s *Z-*test and the Mann–Whitney *U* test combined within the option “Mann–Whitney test” of software package Statistica (Statsoft™, USA).

## Results

### Seven Aggressiveness-Related Genes Chosen for Analysis in This Work

Table 1 presents the seven analyzed aggressiveness-related genes found in PubMed (Lu, 2011)—*Cacna2d3*, *Gad2*, *Gria2*, *Mapk1*, *Nos1*, *Pomc*, and *Syn1*—over- or underexpression of which corresponds to aggressive or domesticated behavior that has the same direction as natural selection as described in subsection “*Study design*.” *Cacna2d3* deficiency causes iron anemia (Baeza-Richer et al., 2015), which is associated with depressive behavior (Kassir, 2017). *Gad2* knockout mice are characterized by sensitized pain behavior (Zhang et al., 2011). It was shown that exposure to fox urine as a fear inducer causes *Gria2* overexpression in mice (Liu et al., 2010). Verhoeven et al. (2011) described the first adolescent patient with a long history of complaints about anxiety and a distal 22q11 microdeletion containing the human *Mapk1* gene, deletion of which can increase the neurobiological susceptibility to anxiety disorders as they concluded. Gammie and Nelson (1999) reported reduced maternal aggression in *Nos1*-deficient female mice. Kiefer et al. (2002) observed statistically significant negative linear correlations between anxiety and POMC levels in alcoholics during the two most adverse days: 1 and 14 days after alcohol withdrawal. A mouse model of human behavior (Park et al., 2014) revealed that early life stress can epigenetically reprogram *Syn1* for underexpression, which causes overanxiety in adulthood.

### Differential Expression of the Seven Analyzed Genes Within the Two Indicator Brain Regions

**Table S** presents our qPCR data on the expression levels of the seven analyzed aggressiveness-related genes in both the hypothalamus and PAG of the aggressive and domesticated male rats. These magnitudes vary from 0.12 to 3.41.

First, we conducted the Kolmogorov–Smirnov test for the normal distribution, the results of which are presented in **Table S**. As one can see, none of the genes being analyzed at the same time follows a normal distribution and shows equality of dispersion of its expression in the aggressive and domesticated male rats being compared. This result justifies the replacement of a pair of traditionally used statistical metrics—mean and SEM (characterizing the assumed normal distribution of experimental values)—by an equivalent pair of statistics, Dn and Up (see the main section “*Materials and methods*”) free of such an assumption. This assumption turned out to be erroneous in the case of our experiment.

**Figure 1:**
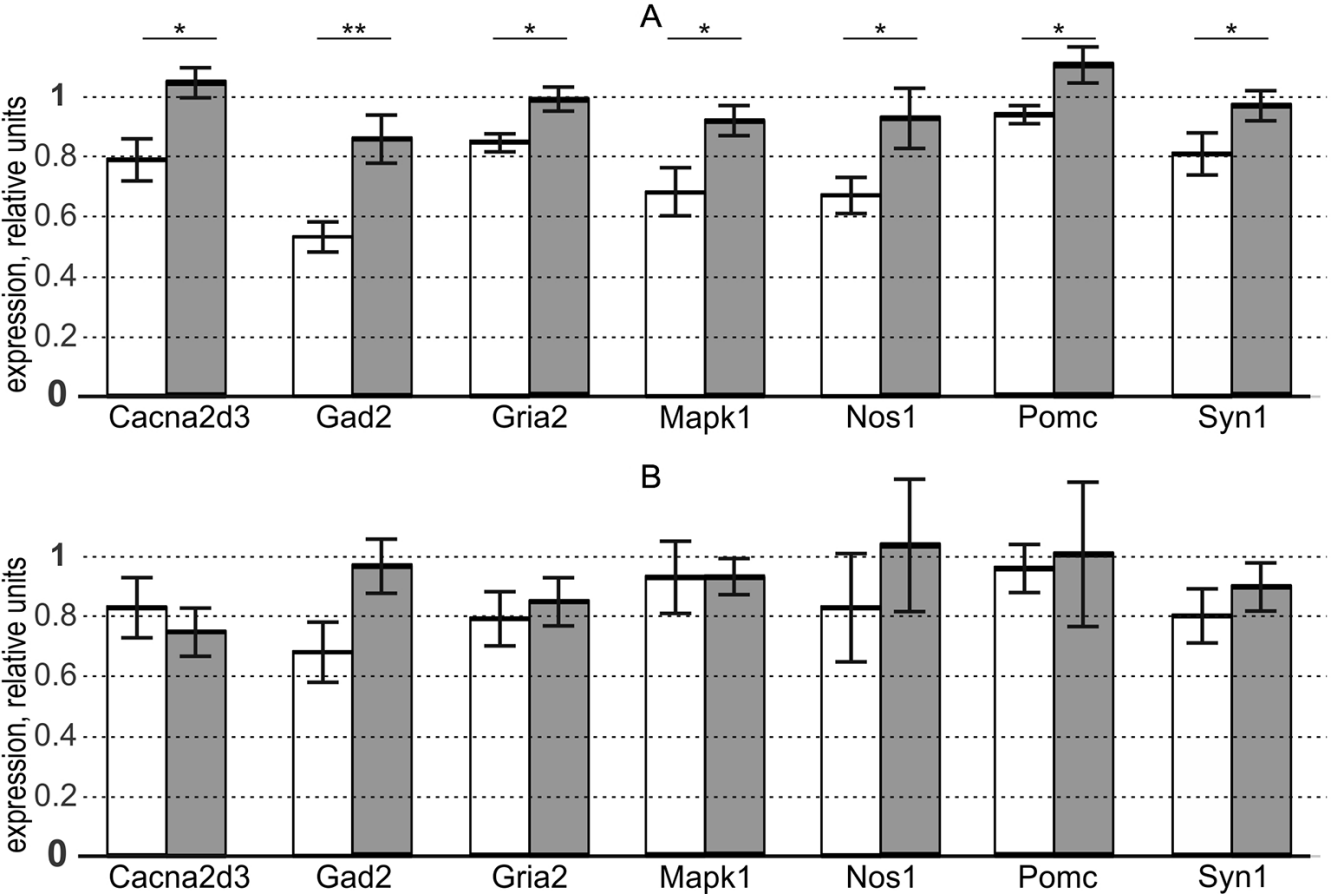
In aggressive male adult rats versus domesticated ones, seven genes selected via the criteria of our study design are statistically significantly overexpressed in the hypothalamus, but not in PAG, where there is no expression of the aggressiveness-related genes in males. white bars, domesticated rats; grey bars, aggressive rats; bar height, mean; error bars, standard error of mean (SEM); Asterisks * and ** denote statistical significance at *p* < 0.05 and *p* < 0.01, respectively. **(A)** hypothalamus (as an indicator of the presence of the aggressiveness-related expression of genes). **(B)** periaqueductal gray (as an indicator of the absence of the aggressiveness-related expression of genes, as a control within the framework of this study). **Table S** presents the gene expression levels revealed by qPCR and their statistical analysis performed in this work (see **Supplementary file** “Supplementary experiment”).

This is why we used the “Mann–Whitney test” option of Statistica (Statsoft™, USA) to test the significance of the overexpression of these genes in the aforementioned brain regions of the aggressive male rats versus domesticated ones. The results are presented in the three rightmost columns of **Table S** and, additionally, in **Figure 1**.

Panel (A) of the figure shows differential expression of the chosen genes in the hypothalamus. As readers can see, we found that the aggressive and domesticated male rats differ in the expression of these genes, in agreement with the rationale for the choice of these genes (**Table 1**) and widespread use of the hypothalamus as a universal behavioral regulator (Walker et al., 2016).

Panel (B) of **Figure 1** reveals the absence of differences between the two groups of male rats (aggressive and domesticated) in the expression of these genes in PAG. This finding is consistent with the specificity of this indicator brain region for both postpartum sexual aggression and maternal aggression (Lonstein and Stern, 1998), which are both absent in the male rats used here (as a control within the framework of this study).

Given that the “Mann–Whitney test” option of Statistica (Statsoft™, USA) additionally carries out Fisher’s *Z*-test, **Table S** contains its results too. Thus, both the nonparametric Mann–Whitney *U* test and parametric Fisher’s *Z*-test independently confirmed our prediction of the overexpression of the analyzed genes in the hypothalamus, but not PAG, of the aggressive male rats versus domesticated ones (see subsection “*Study design*”). This means robustness of the match between our prediction and qPCR data with respect to the variation of the statistical tests.

Using Bonferroni’s correction for multiple tests, we evaluated the statistical significance of the overall result of our study as a whole as follows. Within the approximation of equiprobable positive and negative outcomes (p_0_ = 1/2) of each of the 14 comparisons between our prediction and qPCR data (**Table S**), the probability of the observed or better final result is p_Σ_ = p_0_^14^ = (1/2)^14^ = 2^−14^. Because our final result generalizes N = 56 partial statistical tests (i.e., 28 Kolmogorov–Smirnov tests, 14 Mann–Whitney *U* tests, and 14 Fisher’s *Z-*tests, as shown in **Table S**), Bonferroni’s correction of the statistical significance is p_Bonferroni_ = Np_Σ_ = 56 × 2^-14^ < 100/10000 = 0.01. This result means that the overall findings of our study as a whole are statistically significant too (i.e., we analyzed sufficient and balanced numbers and sufficient diversity of statistical criteria, animals, brain regions, and genes within this project).

## Discussion

On the basis of our prediction of natural selection against underexpression of aggressiveness-related genes (Chadaeva et al., 2017), which was already proved on F1 hybrid mice (Chadaeva et al., 2019), here we predicted and then demonstrated overexpression of *Cacna2d3*, *Gad2*, *Gria2*, *Mapk1*, *Nos1*, *Pomc*, and *Syn1* in the hypothalamus, but not PAG, of aggressive male rats versus domesticated ones. This result means predictability of this phenomenon only within the framework of the basic principle of breeding genetics taken into account together with the natural selection against underexpression of aggressiveness-related genes rather than exceptional importance of these genes for aggressive behavior. For example, many studies show the key roles of genes *5HTT*, *Comp*, *Maoa*, and *Drd4* for manifestation of aggressive behavior. By contrast, overexpression of *Comp* reduces aggressiveness (Wilhelm et al., 2013), whereas either overexpression or underexpression of *5HTT*, depending on the brain region, can either weaken or enhance this trait (Kudryavtseva et al., 2017). Underexpression of genes *Maoa* (Bortolato et al., 2018) and *Drd4* (Keck et al., 2013) can increase it. Consequently, these genes well known for their association with aggressiveness cannot be in our set of analyzed genes (see “*Study Design*” subsection).

According to Bonferroni’s correction for multiple tests, our study as a whole is statistically significant and involves both sufficient and balanced diversity of animals, brain regions, and genes. Indeed, Cushing (2016) exemplified such a qPCR study using the same number of animals as in our study, as did Ilchibaeva et al. (2018) in terms of the total number of the genes being analyzed.

Although many brain regions are studied regarding aggressiveness [see, e.g. Ilchibaeva et al. (2018)], in this work, we chose the hypothalamus–PAG pair as two indicators (“presence” and “absence,” respectively) of the aggressiveness-related expression of genes, to compare their gene expression patterns as did Gorinski et al. (2019) using prefrontal cortex and cerebellum in the case of depression.

Besides, according to the recommendations of Bustin et al. (2009), several reference genes should be used for any quantitative analysis of any gene expression changes during embryogenesis, postnatal ontogenesis, puberty, pregnancy, aging, pathogenesis, and recovery as well as for the early diagnosis of pathologies and for monitoring of treatment efficacy. That is why, without quantitative measures, we qualitatively answered the binary question “Do intergroup differences exceed the intergroup similarities statistically significantly or insignificantly in one animal model of human aggressiveness (**Figure 1**) designed using the fact that this difference is already proven in another model of human aggressiveness (**Table 1**)?” To give an example, wild and domesticated foxes differ in colors (a qualitative trait, not a quantitative trait): red (main) and white (albino) versus red (main) and white (albino) as well as “Black crystal,” “Ermine-like,” “Silver sable-like,” “Amber-gold pastel,” “Steel-blue,” “Platinum,” “Cobalt,” “Purple,” “Pearl,” “Ashen,” “Beige,” “Peach,” “Straw,” and many other commercial fur colors rarely or never seen in the wild (Ponomarenko et al., 2017).

As for the amount of research data on the human genes homologous to the seven rat genes studied here, using these genes and search option “Human,” public database STRING^2^ (von Mering et al., 2005) associated them statistically significantly with long-term depression (p < 0.001), circadian entrainment (p < 0.001), Alzheimer’s disease (p < 0.025), Cushing’s syndrome (p < 0.025), and other health problems. In addition, it associated these genes with the research articles on disorders of the central nervous system during chronic IL-6 overexpression at p < 0.00001 (Gruol et al., 2011) and with articles on stress-caused transcriptome changes at p < 0.001 (Liu et al., 2008), among other research papers. In particular, more than a half (four of seven: *Cacna2d3*, *Mapk1*, P*omc,* and *Syn1)* of underexpression cases found here associate domestication with anxiety, which is the key trait for mutual trust within a human–pet pair, as demonstrated for dogs (Zapata et al., 2016), sheep (Coulon et al., 2014), and guinea pigs (Kaiser et al., 2015). Notably, the original research on sports medicine (Tiric-Campara et al., 2012) related to healthy young boxers, kick boxers, and karate fighters, whose combat intensity increases with their increasing anxiety, indicates that this phenomenon prevents injuries under extreme conditions in the arena until the end of a fight or sparring. Finally, Nobel laureate Daniel Kahneman and his colleagues (Keinan et al., 1999) have shown the importance of anxiety within the framework of Stroop-like interference effects (Stroop, 1935) during human economic decision making under chronic psychological or social stress.

## Conclusion

Altogether, our findings seem to support positive perspectives of further studies on recently discovered domestication syndromes (Theofanopoulou et al., 2017). Consequently, because the current study considered human aggression under the influence of a heuristically limited number of genes that have many additional functions in many other traits, it would be relevant and worthwhile to extend uniformly this gene set to differentially expressed genes (DEGs) in genome-wide transcriptomes for aggressive and domesticated animals (e.g., rats as in this study) compared with each other. In this case, next, the search for the statistically significant similarities between such DEGs of a given aggressive animal versus domesticated animal (in comparison with the DEGs seen in patients with a certain disease versus relatively healthy volunteers) may point to target genes as an answer to the question “Which domesticated animals (and how) can serve as models for which human diseases?” Finally, on this basis, using our public Web service SNP_TATA_Comparator (Ponomarenko et al., 2015), it may be possible to predict candidate SNP markers within these target genes for the above-mentioned diseases in humans as self-domestication syndromes. These computer-based predictions could accelerate the clinical arbitrary search for such genetic markers in cohorts of patients as compared to volunteers. Accordingly, **Table 1** and **Figure 1** suggest that the domesticated male rats studied here represent a candidate animal model of either CACNA2D3-, MAPK1, POMC-, or SYN1-deficient chronic anxiety disorders as human self-domestication syndromes. In this regard, Theodore Dobzhansky (1963) generalized self-domestication–related traits as follows: “man is genetically specialized to be unspecialized.”

## Ethics Statement

The research protocol was approved by the Interinstitutional Commission on Bioethics at the Institute of Cytology and Genetics of the Siberian Branch of the Russian Academy of Sciences (Novosibirsk, Russia).

## Author Contributions

MP wrote the manuscript. SS and RK performed the experiment on rats. NK and IC performed the qPCR experiment. AB contributed to data analysis. ES contributed to corrections in the revised manuscript. DO contributed to the keyword search in Web databases.

## Funding

Manuscript writing was supported by SB RAS Integration Project #0324-2018-0021 (for MP). The use of the equipment at the Center for Genetic Resources of Laboratory Animals at ICG SB RAS was supported by project #RFMEFI62117X0015 from the Russian Ministry of Science and Higher Education and by project #0324-2019-0041 from the Russian Government Budget (for SS). The data compilation was supported by the 5-100 Excellence Programme (for RK). The qPCR experiment was performed on the equipment of the Genomic Research Center at ICG SB RAS that was supported by the project #0324-2019-0042 from the Russian Government Budget (for NK). The concept and study design were supported by grant #18-34-00496 from the Russian Foundation for Basic Research (for IC). The data analysis was supported by projects: #0324-2019-0040 from the Russian Government Budget (for AB). Correction of the revised manuscript was supported by the project “Investigation, analysis and complex independent expertise of projects of the National technological initiatives, including the accompanying of projects of ‘road map’ ‘NeuroNet’,” which is executed within the framework of the state assignment #28.12487.2018/12.1 of the Ministry of Science and Higher Education of the Russian Federation (for ES). The Web database search was supported by the Russian Federal Science & Technology Program for the Development of Genetic Technologies (for DO).

## Conflict of Interest

The authors declare that the research was conducted in the absence of any commercial or financial relationships that could be construed as a potential conflict of interest.

## Footnotes

1
http://wwwmgs.bionet.nsc.ru/cgi-bin/mgs/tatascan/start.pl

2
https://string-db.org/
