# Supplementary material for: A Rat Model of Human Behavior Provides Evidence of Natural Selection Against Underexpression of Aggressiveness-Related Genes in Humans
Source: Front Genet. 2019 Dec 13;10:1267. doi: 10.3389/fgene.2019.01267 (PMC6923764; doi:10.3389/fgene.2019.01267)
Supplement: Supplementary file 1 [file DataSheet_1.pdf]

A rat model of human behavior provides evidence of natural selection against underexpression of aggressiveness-related genes in humans

Dmitry Oshchepkov[1], Mikhail Ponomarenko[1,2], Natalya Klimova[1], Irina Chadaeva[1,2,*], Anatoly Bragin[1], Ekaterina Sharypova[1,2], Svetlana Shikhevich[1], Rimma Kozhemyakina[1,2]

[1]Institute of Cytology and Genetics, Siberian Branch of Russian Academy of Sciences, Novosibirsk 630090, Russia; [2]Natural Science Department, Novosibirsk State University, Novosibirsk 630090, Russia;
***Correspondence:** ichadaeva@bionet.nsc.ru

Supplementary experiment

The experiments were performed on 12 adult male rats (*Rattus norvegicus*) of two outbred lines, which were artificially bred for over 70 generations for aggressive or tame (hereinafter: domesticated) behavior under standard conditions of the Conventional Animal Facility of the Institute of Cytology and Genetics (Novosibirsk, Russia) as described in refs. (Belyaev and Borodin, 1982; Naumenko et al., 1989; Plyusnina and Oskina, 1997).

The total number of rats was 12, namely, six aggressive animals and six domesticated ones, each weighing 250–270 g and 4 months old. All 12 male rats came from different litters and were unrelated. These animals were pair-housed in 43 × 21.5 × 25.5 cm plastic cages and kept on a 12 h:12 h light:dark cycle (lights on at 07:00 h). Food and water were available ad libitum including during testing.

For each rat at the age of 3 months, a standard "glove" test was conducted during the first 4 h of the light phase of the light/dark cycle as described elsewhere (Plyusnina and Oskina, 1997). In short, this behavioral test characterizes the reaction of the rat to the experimenter's hand protected by a thick glove, on a numerical scale from −4, which corresponds to maximum aggressiveness, to +4 as maximum domestication. In Table S, the row "Glove-test" contains the results of this test for each of the 12 male rats under study.

Just after the above "glove" test was completed, all the rats were returned to the standard conditions described above for a period of 1 month until euthanasia and the preparation of the samples of their hypothalamus and periaqueductal gray (PAG) in this work. Using this 1-month delay, we reduced the stress effects of the "glove" test on the results of qPCR (see the main section "Materials and Methods" of this work).

All the above procedures were in line with Directive 2010/63/EU of the European Parliament and of the Council of September 22, 2010, on the protection of animals used for scientific purposes. Manipulations with animals and experimental procedures were carried out in accordance with the international rules according to the "Guidelines for the care and use of mammals in neuroscience and behavioral research"[1] of 1992. The research protocol was approved by the Interinstitutional Commission on Bioethics at the Institute of Cytology and Genetics of the Siberian Branch of the Russian Academy of Sciences, 10 Lavrentyev Avenue, Novosibirsk, 630090, Russia.

References

Belyaev, D.K., and Borodin, P.M. (1982) The influence of stress on variation and its role in evolution. *Biol. Zent. Bl*. **100**: 705–714.

Naumenko, E.V., Popova, N.K., Nikulina, E.M., Dygalo, N.N., Shishkina, G.T., Borodin, P.M. et al. (1989) Behavior, adrenocortical activity, and brain monoamines in Norway rats selected for reduced aggressiveness towards man. *Pharmacol Biochem Behav*. **33**, 85–91. doi:10.1016/0091-3057(89)90434-6

Plyusnina, I., and Oskina, I. (1997) Behavioral and adrenocortical responses to open-field test in rats selected for reduced aggressiveness toward humans. *Physiol Behav*. **61**, 381–385. doi:10.1016/S0031-9384(96)00445-3

---

[1]https://grants.nih.gov/grants/olaw/National_Academies_Guidelines_for_Use_and_Care.pdf

Table S. The qPCR data on the expression of analyzed genes within both the hypothalamus and periaqueductal gray (PAG) of the aggressive and domesticated rat males and statistical analysis of these data obtained in this study

Study design		Behavioral "glove" test and the qPCR data on gene expression (this work)												Output of Statistica (Statsoft[TM], USA)					
Set	Rat male #	1		2		3		4		5		6		Kolmogorov–Smirnov test for normal distribution, $N(M_0, \sigma^2)$			Fisher`s Z-test and Mann–Whitney *U* test		
D	"Glove" test	3.00		3.00		3.00		3.00		3.00		3.00							
A		−3.00		−3.00		−3.00		−3.00		−3.00		−3.00							
	Gene (OMIM ID)	Dn	Up	Dn	Up	Dn	Up	Dn	Up	Dn	Up	Dn	Up	$M_0 \pm$ SEM	d	p	U	Z	p
	Hypothalamus:																		
D	*Cacna2d3*	0.59	0.66	0.85	1.10	1.01	1.23	0.94	1.02	0.41	0.57	0.59	0.68	0.80 ± 0.07	0.18	0.26	**32**	**2.25**	**0.05**
A	(606399)	0.86	0.91	0.95	1.16	0.88	0.99	0.92	1.11	1.10	1.32	1.17	1.30	1.06 ± 0.04	0.16	0.13			
D	*Gad2*	0.41	0.53	0.47	0.50	0.59	0.67	0.36	0.51	0.70	0.95	0.32	0.42	0.54 ± 0.05	0.18	0.23	**26**	**2.59**	**0.01**
A	(138275)	0.88	1.11	0.39	0.44	0.87	1.17	0.52	0.82	0.95	1.10	1.04	1.12	0.87 ± 0.08	0.18	0.24			
D	*Gria2*	0.66	0.74	0.83	0.94	0.85	1.11	0.88	0.92	0.82	0.93	0.75	0.86	0.86 ± 0.03	0.15	0.10	**33**	**2.19**	**0.05**
A	(138247)	0.88	1.13	0.76	0.84	0.98	1.04	0.99	1.02	0.82	1.27	1.11	1.15	1.00 ± 0.04	**0.11**	**0.01**			
D	*Mapk1*	0.31	0.37	0.88	1.06	0.82	1.1	0.59	0.91	0.5	0.72	0.3	0.76	0.69 ± 0.08	**0.12**	**0.05**	**34**	**2.16**	**0.05**
A	(176948)	0.96	0.98	0.6	0.67	0.94	1.08	0.83	1.19	0.95	1.09	0.82	1.06	0.93 ± 0.05	0.18	0.27			
D	*Nos1*	0.38	0.49	0.55	0.65	0.76	0.99	0.79	1.02	0.39	0.62	0.69	0.77	0.68± 0.06	**0.12**	**0.05**	**37**	**1.99**	**0.05**
A	(608226)	1.33	1.54	0.48	0.78	0.94	1.16	0.68	0.98	0.54	0.75	1.03	1.09	0.94 ± 0.10	**0.11**	**0.01**			
D	*Pomc*	0.82	0.88	0.85	0.93	0.94	1.13	0.94	1.03	0.88	0.91	1.03	1.08	0.95 ± 0.03	0.21	0.43	**31**	**2.34**	**0.05**
A	(176830)	1.24	1.67	0.99	1.22	0.86	0.95	0.98	1.09	0.99	1.02	1.15	1.33	1.12 ± 0.06	0.18	0.24			
D	*Syn1*	0.56	0.57	0.88	1.00	0.70	1.31	0.89	1.13	0.72	0.78	0.61	0.63	0.82 ± 0.07	0.15	0.11	**37**	**1.96**	**0.05**
A	(313440)	0.82	1.18	0.59	0.83	1.00	1.23	1.01	1.02	0.91	1.15	0.98	1.08	0.98 ± 0.05	0.15	0.12			
	Periaqueductal gray (PAG):																		
D	*Cacna2d3*	0.41	0.61	0.92	1.10	1.11	1.15	0.86	0.97	0.31	0.34	0.99	1.25	0.84 ± 0.10	0.19	0.32	59	0.69	0.48
A	(606399)	0.51	0.57	0.91	1.09	0.68	0.90	0.97	1.36	0.46	0.51	0.53	0.65	0.76 ± 0.08	0.19	0.32			
D	*Gad2*	0.38	0.61	0.53	1.04	0.12	1.38	0.39	0.46	0.87	1.07	0.60	0.80	0.69 ± 0.10	0.16	0.17	40	1.81	0.07
A	(138275)	1.03	1.11	0.81	0.92	1.04	1.38	0.79	0.95	0.4	0.52	1.33	1.45	0.98 ± 0.09	**0.11**	**0.01**			
D	*Gria2*	0.65	0.75	0.39	1.61	0.70	1.07	0.56	0.66	0.88	0.99	0.65	0.73	0.80 ± 0.09	0.23	0.54	59	0.72	0.47
A	(138247)	1.27	1.39	0.71	0.85	0.96	1.08	0.68	0.88	0.48	0.58	0.69	0.75	0.86 ± 0.08	0.15	0.11			
D	*Mapk1*	0.55	0.96	1.28	1.96	0.68	1.26	0.63	0.81	0.55	0.84	0.77	0.99	0.94 ± 0.12	0.20	0.35	62	0.54	0.58
A	(176948)	1.14	1.26	0.96	1.11	1.06	1.10	0.78	0.94	0.58	0.61	0.78	0.98	0.94 ± 0.06	0.16	0.14			
D	*Nos1*	0.27	0.39	1.43	2.34	0.79	1.29	0.31	0.97	0.36	0.40	0.72	0.85	0.84± 0.18	0.18	0.25	65	0.37	0.70
A	(608226)	1.14	1.38	0.21	1.79	2.00	2.48	0.76	1.20	0.17	0.28	0.45	0.68	1.05 ± 0.22	0.14	0.07			
D	*Pomc*	1.01	1.05	1.29	1.32	0.96	1.11	0.32	0.69	0.93	1.07	0.88	1.05	0.97 ± 0.08	0.19	0.32	63	0.49	0.62
A	(176830)	0.9	1.02	0.36	0.83	0.15	0.41	0.56	3.41	1.03	1.18	1.12	1.31	1.02 ± 0.24	0.28	0.75			
D	*Syn1*	0.64	0.74	0.38	1.30	0.89	1.40	0.54	0.59	0.68	0.78	0.86	0.97	0.81 ± 0.09	0.14	0.08	58	0.75	0.45
A	(313440)	1.22	1.39	0.77	0.96	1.00	1.20	0.58	0.66	0.57	0.63	0.89	1.02	0.91 ± 0.08	0.15	0.09			

Genes: *Cacna2d3*, Ca-channel α2δ3 subunit; *Gad2*, glutamate decarboxylase 2; *Gria2*, glutamate receptor 2; *Mapk1*, mitogen-activated protein kinase 1; *Nos1*, NO synthase 1; *Pomc*, proopiomelanocortin; *Syn1*, synapsin 1. **Sets:** D and A as domesticated rats and aggressive rat males, respectively; Dn and Up as the lower and upper boundaries of the [mean ± standard deviation] interval of the qPCR data, respectively. **Output of the package Statistica (Statsoft™, USA)**: $N(M_0, \sigma^2)$ as the normal distribution (here: M_0 and σ as mean and standard deviation, accordingly); $SEM = \sigma/(n)^{1/2}$ as standard error of mean (here: n = 12 as amount of qPCR data under consideration); d, U, Z, and p as the *d*-value of the Kolmogorov–Smirnov test for normal distribution, *U*-value of the Mann–Whitney *U* test, *Z*-values of Fisher's *Z*-test (output of the "Mann–Whitney test" option in the software used) and *p* value of statistical significance, respectively. Statistically significant data are **boldfaced**.
